# CD40mAb adjuvant induces a rapid antibody response that may be beneficial in post-exposure prophylaxis

**DOI:** 10.1186/1476-8518-8-1

**Published:** 2010-02-04

**Authors:** Vijay NS Bhagawati-Prasad, Evy De Leenheer, Nadine P Keefe, Lorna A Ryan, Jennifer Carlring, Andrew W Heath

**Affiliations:** 1 Department of Infection and Immunity, University of Sheffield Medical School, Beech Hill Rd, Sheffield S10 2RX, UK; 2 Adjuvantix Ltd, c/o Fusion plc, Sheffield Bioincubator, Leavygreave Rd, Sheffield, UK

## Abstract

Active vaccination can be effective as a post-exposure prophylaxis, but the rapidity of the immune response induced, relative to the incubation time of the pathogen, is critical. We show here that CD40mAb conjugated to antigen induces a more rapid specific antibody response than currently used immunological adjuvants, alum and monophosphoryl lipid A™.

## Findings

Post-exposure prophylaxis (PEP), or the induction of protection against an infectious disease after exposure to the pathogen, is either utilised or has been suggested as an appropriate course of action for a number of diseases, including rabies [1], anthrax [2], varicella [3,4], HIV and Hepatitis A [5]. PEP can be divided into three categories: The administration of antibiotics or antivirals, passive immunization using specific immunoglobulin, and active immunization (vaccination).

In some cases appropriate antimicrobial chemotherapy may not be available, or there may be a worry that the pathogen could be resistant to the agent, particularly in bioterrorism cases. Passive immunization using immunoglobulin may be a suitable alternative, if the pathogen is susceptible to antibody-mediated killing. However, active vaccination has the potential advantages of lower cost, less risk of adventitious pathogen transfer, and most importantly the induction of long-term protection. The use of active vaccination as PEP however depends upon the rapidity with which a protective immune response can be generated, in comparison with the incubation period of the pathogen post-exposure. The kinetics of the immune response are therefore a potential rate-limiting step for the efficacy of post-exposure vaccination.

Immune responses against vaccines are enhanced by immunological adjuvants. Aluminum salts are the only widely licensed immunological adjuvants [6], but the adjuvant monophosphoryl lipid A (MPL™) is now licensed in some countries for use in the cervical cancer vaccine, Cervarix™ [7] and may shortly be licensed for use in a wider range of vaccines. Aside from MPL™, there is a large amount of research ongoing into other potential adjuvants, including host co-stimulatory molecules [8], TLR agonists [6,9], other particulate carriers [10,11] and combinations of these approaches [12,13].

Agonistic antibodies against the antigen presenting cell surface antigen CD40 are able to mimic the effect of binding of the ligand, CD154, both *in vitro *[14] and *in vivo *[15]. We have shown that agonistic CD40monoclonal antibody (mAb) is an effective immunological adjuvant at low doses when chemically conjugated to antigen. It is able to enhance antibody [16,17] as well as T helper responses [18]. CD40 antibody or ligand is also being investigated in cancer therapy and vaccination [19,20]. As we believe CD40mAb conjugate acts as an adjuvant at least in part *via *a direct effect on B cells [21], we were interested in assessing the rapidity of the antibody response induced by CD40mAb in comparison with other adjuvants. We used the model antigen, ovalbumin, in order to compare the kinetics of the induced antibody response between CD40 conjugate, MPL™ and the widely used alum adjuvant.

Female C57Bl/6 mice aged 6-8 weeks were obtained from Harlan UK Ltd and housed in accordance with strict Home Office guidelines. Ovalbumin (Sigma) was conjugated to the CD40mAb 10C8 [22] as previously described [16]. Four groups of 15 mice were immunised intraperitoneally with either 10 μg of conjugate, 10 μg of ovalbumin either alone, with MPL™ (10 μg, Sigma) or adsorbed onto Aluminium hydroxide (200 μg, 5). Five of the 15 mice were bled every 3 days in rotation, and anti-ovalbumin IgG endpoint titers determined by ELISA assay as previously described [16]

**Figure 1:**
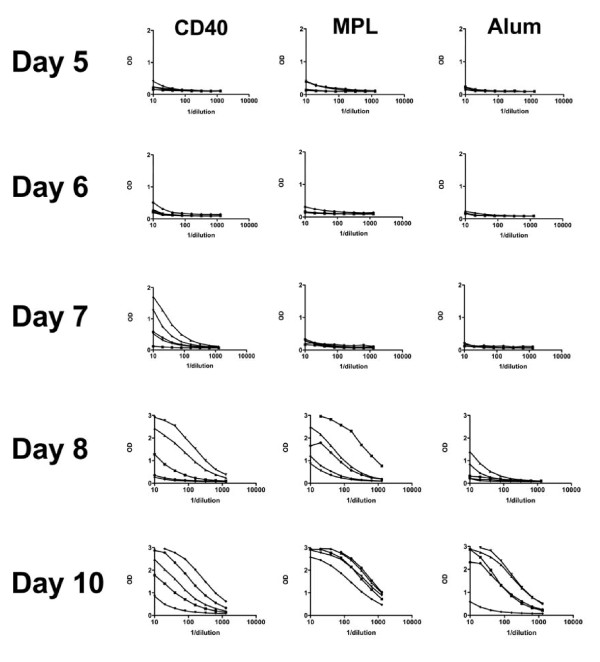
**C57Bl/6 mice were immunized once, as described in the text, with ovalbumin and the adjuvant shown at the top of the figure, and bled at various days post-immunization (shown on left side of figure)**. ELISA data are plotted for 5 mice in each case with the reciprocal of the serum dilution on the x axis, and optical density at 490 nm on the y axis. Note the scale of the y axis varies by row. Response to CD40mAb adjuvanted vaccine was significantly better than the response to MPL or alum at day 7 (p < 0.05, One-way ANOVA with Dunnet's post-test).

ELISA results are shown in Fig 1. CD40mAb conjugate induced an IgG response against OVA by day 7 post immunization, whereas no IgG response to OVA+MPL™ or OVA+alum was seen until day 8, and in the case of alum this was weak. The results shown are representative of a total of three experiments. Total immunoglobulin responses (including IgM) tended to arise a day earlier, but showed the same difference in kinetics between CD40 and the other two adjuvants.

CD40mAb-OVA conjugate induces a more rapid IgG response in mice than either the established adjuvant, alum, or the newer adjuvant, MPL™. How much faster the response to a CD40mAb vaccine versus an MPL adjuvanted vaccine would be in humans would need to be determined empirically. How important a more rapid response would be would depend upon the titers required to protect against a particular pathogen, as well as the window of opportunity available to prevent disease. We propose that CD40mAb conjugates may have utility in post-exposure prophylaxis when a rapid antibody response is desirable.

## Abbreviations used

MPL: monophosphoryl lipid A; TLR: Toll like receptor; ELISA: Enzyme linked immunosorbent assay; PEP: post-exposure prophylaxis; mAb: monoclonal antibody.

## Competing interests

AH is a Director of Adjuvantix Ltd and also holds some stock in Adjuvantix. Adjuvantix Ltd have an interest in CD40mAb based immunological adjuvants.

## Authors' contributions

VB, ED, NK and LR performed the experimental work. Experiments were designed by JC, ED and AH. AH, ED and JC wrote the manuscript.

All authors have read and approved the final manuscript.
